# Improvements in naturalistic speech-in-noise comprehension in middle-aged and older adults after 3 weeks of computer-based speechreading training

**DOI:** 10.1038/s41539-023-00179-6

**Published:** 2023-09-04

**Authors:** Raffael Schmitt, Martin Meyer, Nathalie Giroud

**Affiliations:** 1 Department of Computational Linguistics, University of Zurich, Zurich, Switzerland; 2 International Max Planck Research School on the Life Course: Evolutionary and Ontogenetic Dynamics (LIFE), Zurich, Switzerland; 3 Language & Medicine Centre Zurich, Competence Centre of Medical Faculty and Faculty of Arts and Sciences, University of Zurich, Zurich, Switzerland; 4 Department of Comparative Language Science, University of Zurich, Zurich, Switzerland; 5 Center for the Interdisciplinary Study of Language Evolution (ISLE), University of Zurich, Zurich, Switzerland; 6 Cognitive Psychology Unit, Alpen-Adria University, Klagenfurt, Austria; 7 Neuroscience Center Zurich, University of Zurich and ETH Zurich, Zurich, Switzerland

**Keywords:** Human behaviour, Human behaviour

## Abstract

Problems in understanding speech in noisy environments are characteristic for age-related hearing loss. Since hearing aids do not mitigate these communication problems in every case, potential alternatives in a clinical rehabilitation plan need to be explored. This study investigates whether a computer-based speechreading training improves audiovisual speech perception in noise in a sample of middle-aged and older adults (*N* = 62, 47–83 years) with 32 participants completing a speechreading training and 30 participants of an active control group completing a foreign language training. Before and after training participants performed a speech-in-noise task mimicking real-life communication settings with participants being required to answer a speaker’s questions. Using generalized linear mixed-effects models we found a significant improvement in audiovisual speech perception in noise in the speechreading training group. This is of great relevance as these results highlight the potential of a low-cost and easy-to-implement intervention for a profound and widespread problem as speech-in-noise comprehension impairment.

## Introduction

Hearing loss is the most prevalent sensory impairment in older adults^1^ with its prevalence being expected to grow with the ageing of the global population^2^. A common strategy in aural rehabilitation is fitting patients with hearing aids. However, despite tremendous technological advances in recent years, hearing aids do not provide relief in every clinical case of hearing loss^3^. It is to be expected, for example, that in an older adult with speech-in-noise comprehension problems whose hearing threshold is normal, amplification of the acoustic signal will not produce the same results as in a person experiencing the same problems due to poor audibility. For these very cases other interventions are needed, with research showing a shift towards training-based rehabilitation measures^4^.

Leaving hearing loss untreated has serious consequences: in addition to communication problems, which are in themselves disabling for the affected person, hearing loss shows a link with depression^5^, reduced quality of life^6^, and increased risk for dementia^7^. Any form of intervention that counteracts this risk factor can therefore be considered a relevant support for healthy aging.

Besides the acoustic signal, listeners rely on additional sensory cues such as information transported by the interlocutor’s face—information that gains importance in certain situations. When the acoustic signal is degraded by internal (e.g., hearing loss) or external factors (e.g., noise) seeing a speaker’s face can aid speech perception both in normal hearing and hearing-impaired adults (e.g.,^8–10^). It is therefore not surprising that training the use of such visual cues through speechreading training depicts a complementary measure in some clinical rehabilitation plans^11^. Speechreading trainings aim to focus the listeners attention to visual speech cues and complement the degraded acoustic signal. However, research to date paints an ambiguous picture of the effectiveness of such interventions, which is mainly due to methodological factors. Studies addressing potential benefits of speechreading trainings in adults are limited to small sample sizes, lack an adequate control group, or use outcome measures unlikely to be transferable to everyday listening performance^12–15^.

Taking these drawbacks into account, the main goal of the present study was to investigate whether a self-guided computer-based speechreading training improves audiovisual speech perception in noise in a sample of 62 middle-aged and older adults with varying degrees of hearing loss (i.e., pure-tone average (PTA) over 0.5–8 kHz between 10 and 83 dB HL). For this purpose, we compared speech comprehension in noise between a group completing three weeks of speechreading training (ST) and an active control group (AC) that trained a foreign language for three weeks. Due to its indisputable superiority over a passive control, we included an AC in our study. In addition to test-retest effects, the AC controls for possible effects that may arise from adherence to a training schedule and, if cleverly selected, for potential expectancy effects (e.g.,^16^,). Controlling for the latter is however complicated, as participants must receive an intervention that is as close as possible to that of the experimental group and has credible positive effects on the outcome of interest. This was the main reason the AC went through foreign language training as the cover story proclaimed that the study’s aim was to investigate possible effects of language training for audiovisual speech perception in noise.

At pre- and post-training, participants performed an audiovisual speech perception in noise task that served as primary outcome measure. As opposed to previous studies, we refrained from using commonly used tasks such as sentence repetition, target word detection, or consonant identification, as our aim was to measure speech comprehension in a way that mimics real-word communication where comprehending the meaning of an utterance is paramount. For this purpose, participants saw a female speaker on screen that asked questions one could encounter in everyday life. Participants were instructed to give an answer vocally into a microphone that clearly indicated whether they understood the question or not. Complementary to the objectively measured speech comprehension, we assessed participants’ subjective hearing by means of questionnaires to capture potential training effects in self-perceived everyday listening effort and abilities. To control for potential confounders, participants completed cognitive tasks measuring visual working memory and processing speed, as these two capacities are considered to be key predictors of audiovisual speech comprehension^17^.

In summary, the present study investigates the effect of a speechreading training in a sample of middle-aged and older adults using realistic listening conditions being more representative of real-life communication. We hypothesize that speechreading training has a positive effect, which is reflected in an improvement in audiovisual speech perception in noise and subjectively rated hearing abilities.

## Results

### Group comparisons

**Fig. 1:**
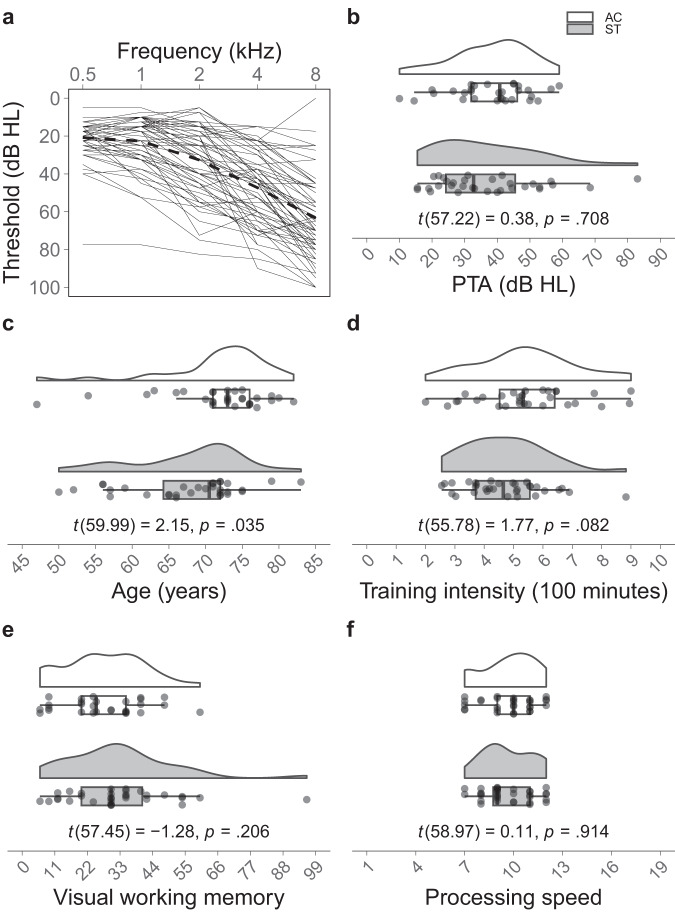
Sample characteristics of the two groups. **a** Hearing threshold for frequencies from 0.5 to 8 kHz. Each line represents an individual participant. The grand average hearing threshold is marked as dotted line. **b** Pure tone average (PTA), **c** age, **d** training intensity, and cognitive abilities in **e** visual working memory and **f** processing speed for each group. T-statistics are shown at the bottom of each plot. Each dot represents an individual participant. Thick lines inside boxplots mark the median. AC active control, ST speechreading training.

Using Welch’s *t*-tests we compared sample characteristics between the two groups to ensure an adequate match. Groups did not differ significantly in terms of PTA (*t*(57.22) = 0.38, *p* = 0.708), training intensity (*t*(55.78) = 1.77, *p* = 0.082), visual working memory (*t*(57.45) = − 1.28, *p* = 0.206), or processing speed (*t*(58.97) = 0.11, *p* = 0.914). However, the AC was significantly older than the ST group (*t*(59.99) = 2.15, *p* = 0.035). Since we included age as a covariate in all subsequent analyses, we argue that potential confounding effects are mitigated. Note that although the magnitude of the effect for training intensity is comparable to the effect of age (i.e., Cohen’s *d* for Welch’s *t*-test: *d*_Age_ = 0.55; *d*_TrainingIntensity_ = 0.45), we did not include training intensity in subsequent analyses. Firstly, we wanted to avoid over-specified regression models, and secondly, the measured training intensity for AC is only an approximation of the actual training duration (see section *Language training*). Summary statistics are listed in Table 1 and plotted in Fig. 1.

**Table 1:** Demographic information and summary statistics by training group.

	Speechreading training	Active control
	*n* = 32 (16 female)	*n* = 30 (16 female)
Hearing threshold (PTA)		
Mean (*SD*)	36.73 (16.05)	38.08 (11.99)
Median [min, max]	32.75 [15.5, 83]	40.75 [10, 59]
Age (years)		
Mean (*SD*)	67.53 (7.82)	71.70 (7.42)
Median [min, max]	70.50 [50, 83]	73.00 [47, 82]
Training intensity (minutes)		
Mean (*SD*)	471.78 (140.35)	543.10 (173.84)
Median [min, max]	465.00 [255, 883]	533.00 [200, 900]
Visual working memory		
Mean (*SD*)	32.88 (18.12)	27.62 (13.87)
Median [min, max]	30.00 [6, 96]	25.00 [6, 60]
Processing speed		
Mean (*SD*)	9.66 (1.54)	9.70 (1.64)
Median [min, max]	9.00 [7, 12]	10.00 [7, 12]

### No significant predictor for visual enhancement

**Fig. 2:**
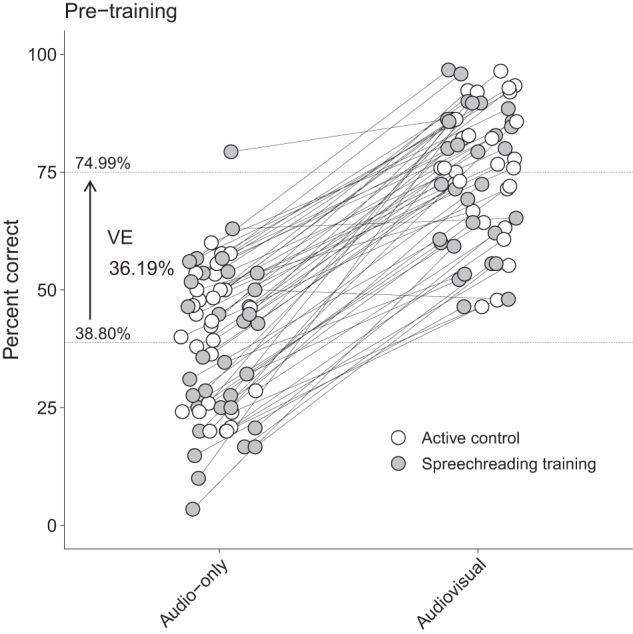
Speech-in-noise comprehension scores at pre-training. The average comprehension scores are shown for both audio-only and audiovisual. Grand averages are depicted above the two dotted lines for the audio-only and the audiovisual condition. Each dot represents an individual participant. VE visual enhancement.

We then investigated the relationship between visual enhancement (VE) (Fig. 2) and several variables. Using multiple regression analysis, none of the predictors showed a significant association with VE (PTA, *β* = 1.81, 95% CI[−4.13, 7.76], *p* = 0.544; age, *β* = −5.07, 95% CI[−11.42, 1.28], *p* = 0.115; visual working memory, *β* = 0.82, 95% CI[−4.66, 6.30], *p* = 0.767; processing speed, *β* = 0.09, 95% CI[−5.56, 5.74], *p* = 0.975) (Supplementary Fig. 1).

### Training effects

#### Speechreading training improves audiovisual speech perception in noise

**Fig. 3:**
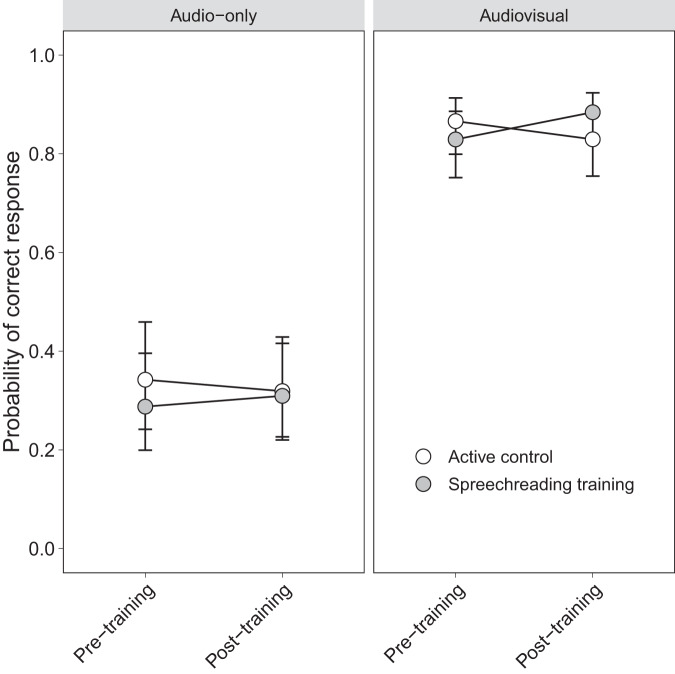
Training effect on speech-in-noise comprehension. Model predictions for the significant three-way interaction between condition, session, and group from the generalized linear mixed-effects model. The speechreading training group shows an improvement from pre- to post-training in the audiovisual condition. Bars depict 95% confidence intervals.

Using generalized linear mixed-effects models (GLMMs) on single-trial data, we found a significant training effect, with the ST group improving significantly from pre- to post-training in the audiovisual (AV) condition. As indicated by the significant three-way interaction between condition, session, and group (Δ*Χ*^2^(4) = 13.62, *p* = 0.009), this effect was only present in the ST group and only for the AV but not the audio-only (A-only) condition (Table 2; Fig. 3). Although not of primary interest, the variance estimates of the random effects in Table 2 suggest that the between-item variance (i.e., τ_00 item_) explains a large portion of the total variance. This underlines the importance of accounting for item variability in the random effects structure when naturalistic stimuli are used.

**Table 2:** Parameter estimates for the effects of condition, session, and group on performance in the speech comprehension task.

Predictors		Odds Ratios	CI	*p*
(Intercept)		1.64	1.13–2.36	**0.009**
condition [AV]		3.57	3.15–4.04	**<0.001**
session [post]		1.02	0.94–1.10	0.606
group [ST]		0.98	0.80–1.21	0.875
age_z		0.92	0.74–1.14	0.459
condition [AV] : session [post]		1.02	0.95–1.10	0.580
condition [AV] : group [ST]		1.06	0.97–1.16	0.202
session [post] : group [ST]		1.13	1.05–1.21	**0.002**
condition [AV] : session [post] : group [ST]		1.07	1.00–1.14	**0.047**
Random effects				
*σ*^2^		3.29		
*τ*_00 subject_		0.58		
*τ*_00 item_		2.88		
*τ*_11 subject.condition[AV]_		0.06		
*τ*_11 subject.session[post]_		0.02		
*τ*_11 item.condition[AV]_		0.16		
*τ*_11 item.session[post]_		0.02		
Contrasts				
condition [A]	−1			
condition [AV]	1			
session [pre]	−1			
session [post]	1			
group [AC]	−1			
group [ST]	1			

Interactions are indicated by the symbol “:”. GLMM configuration: response ~ 1 + condition * session * group + age_z + (1 + condition + session | subject) + (1 + condition + session | item).

Bold values indicates statistical significant *P* values (*P* < 0.05).

**Fig. 4:**
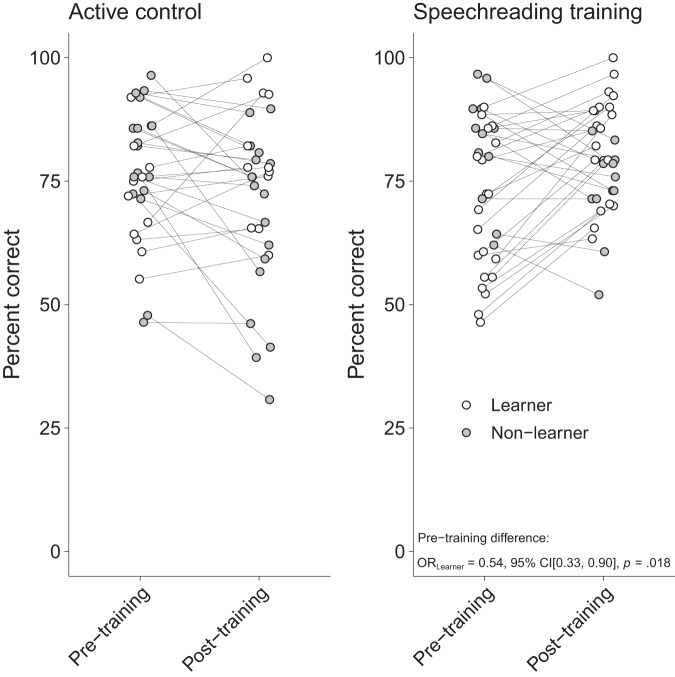
Average audiovisual scores at pre-training. The average audiovisual comprehension scores at pre-training are shown for both learners and non-learners. Learners and non-learners differed significantly in the pre-training score in both active control and speechreading training group. Results from a generalized linear mixed-effects model suggesting learners to perform worse at pre-training than non-learners are depicted at the bottom of the right plot. Each dot represents an individual participant.

**Fig. 5:**
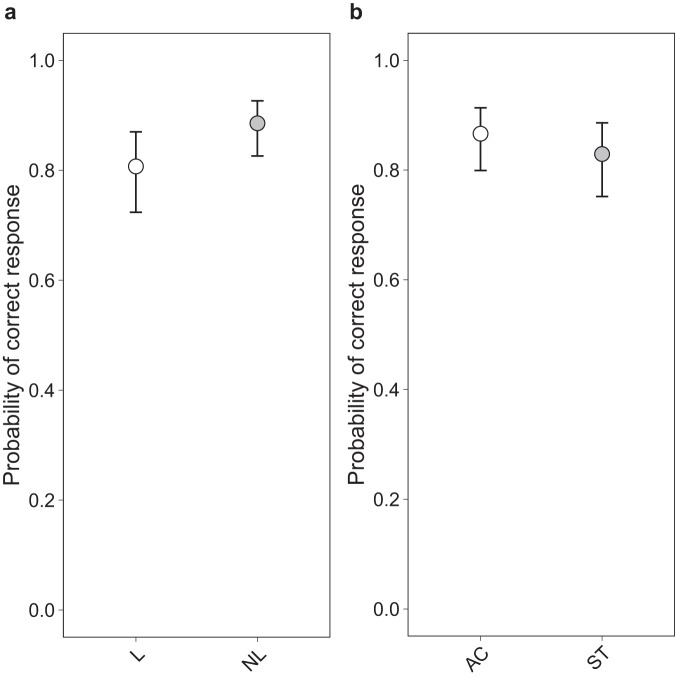
Differences in audiovisual speech comprehension at pre-training. Model predictions for performance differences in audiovisual speech comprehension at pre-training for (**a**) learners and non-learners and (**b**) active control and speechreading training group. Learners showed a significantly lower score at pre-training than non-learners. This difference was not significant between active control and speechreading training group, suggesting that baseline differences did not pose a bias in the observed training effect. Bars depict 95% confidence intervals. L learners, NL non-learners, AC active control, ST speechreading training.

As often seen in clinical trials, baseline and change scores are inherently correlated^18^—a circumstance also present in our data. As can be seen in Figs. 4 and 5 (and Supplementary Table 2), participants improving from pre- to post-training in AV (i.e., *learners*) scored significantly lower at pre-training compared to participants who showed no change or even deteriorated in their performance (i.e., *non-learners*). We complemented the initial GLMM with further analyses to assure that differences in pre-training scores did not confound the observed training effects in ST. To do so, we compared the scores obtained at pre-training for AV between AC and ST. We reran the GLMM described in Table 2 but changed from effect to treatment coding with “AV” in condition, “pre-training” in session, and “AC” in group as reference levels. This way, the main effect of group could be interpreted as difference between AC and ST in AV at pre-training. Indeed, the two groups did not significantly differ at pre-training (OR = 0.75, 95% CI[0.45, 1.25], *p* = 0.271) (Fig. 5 and Supplementary Table 3) suggesting that baseline differences in AV did not pose a potentially confounding factor.

In a further step, we explored whether other variables might underly the observed training effects in the ST group. Using multiple regression, we determined whether the individual learning rate was modulated by PTA, age, hearing aid use, training intensity, visual working memory, or processing speed. Although information gets lost by averaging trial-level data to form a mean learning rate, we chose this analytic approach as this way variables could be tested in one single model. Otherwise, a model would have had to be estimated for each individual variable with the interaction between the respective variable and session. None of the variables showed a significant association with learning rate (PTA, *β* = −0.91, 95% CI[−7.99, 6.18], *p* = 0.794; age, *β* = 0.29, 95% CI[−6.99, 7.56], *p* = 0.936; hearing aid use, *β* = 2.49, 95% CI[−5.98, 10.96], *p* = 0.550; training intensity, *β* = 2.18, 95% CI[−4.46, 8.82], *p* = 0.505; visual working memory, *β* = −1.60, 95% CI[−6.77, 3.58], *p* = 0.531; processing speed, *β* = −1.42, 95% CI[−7.83, 4.99], *p* = 0.653) (Supplementary Fig. 2).

#### Speechreading training does not improve subjective hearing

**Fig. 6:**
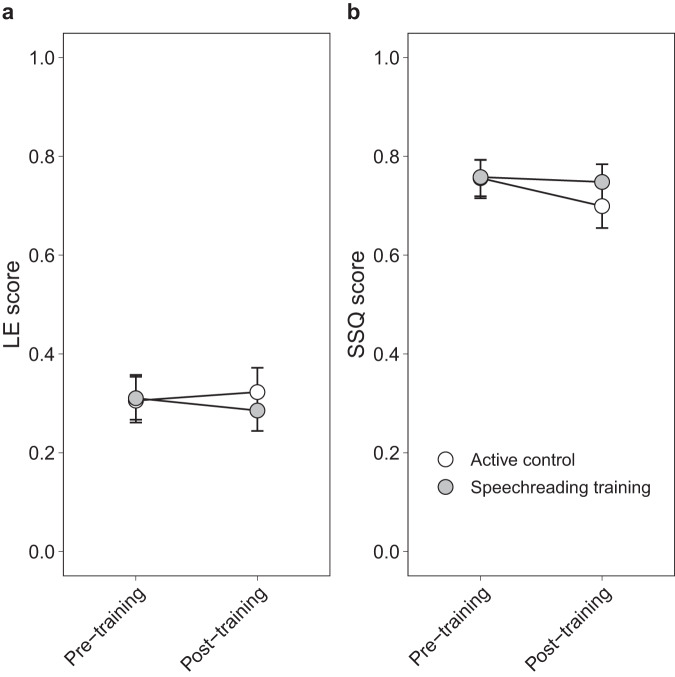
Training effect on subjective hearing. Model predictions for the non-significant two-way interaction between session and group from the generalized linear mixed-effects models. The speechreading training group did not show a significant improvement in subjective hearing from pre- to post-training, neither in (**a**) the Listening Effort Questionnaire nor (**b**) the Speech, Spatial and Qualities of Hearing Scale. Bars depict 95% confidence intervals. LE Listening Effort Questionnaire, SSQ Speech, Spatial and Qualities of Hearing Scale.

In a next step, we investigated whether the observed training effects would translate to subjectively perceived hearing abilities in everyday life. Using GLMMs with a beta distribution, the potential training effect, depicted by the interaction between session and group, was not significant—neither for the perceived listening effort (Δ*Χ*^2^(1) = 2.92, *p* = 0.087) (Fig. 6; Table 3) nor the speech, spatial, and hearing qualities (Δ*Χ*^2^(1) = 3.49, *p* = 0.062) (Fig. 6; Table 4).

**Table 3:** Parameter estimates for the effects of session and group on subjective hearing in the Listening Effort Questionnaire (LE).

Predictors		Odds Ratios	CI	*p*
(Intercept)		0.44	0.38–0.51	**<0.001**
session [post]		0.99	0.94–1.05	0.735
group [EG]		0.96	0.83–1.11	0.598
spta_z		1.55	1.32–1.81	**<0.001**
age_z		0.83	0.71–0.97	**0.023**
session [post] : group [EG]		0.95	0.90–1.01	0.084
Random effects				
σ^2^		0.03		
τ_00 subject_		0.25		
Contrasts				
session [pre]	−1			
session [post]	1			
group [AC]	−1			
group [ST]	1			

Interactions are indicated by the symbol “:”. GLMM configuration: score ~ 1 + session * group + pta_z + age_z + (1 | subject). .

Bold values indicates statistical significant *P* values (*P* < 0.05).

**Table 4:** Parameter estimates for the effects of session and group on subjective hearing in the Speech, Spatial and Qualities of Hearing Scale (SSQ).

Predictors		Odds Ratios	CI	*p*
(Intercept)		2.85	2.51–3.24	**<0.001**
session [post]		0.92	0.86–0.98	**0.006**
group [EG]		1.07	0.93–1.22	0.339
pta_z		0.75	0.65–0.86	**<0.001**
age_z		1.13	0.97–1.31	0.116
session [post] : group [EG]		1.06	1.00–1.13	0.058
Random effects				
* σ*^2^		−0.01		
* τ*_00 subject_		0.20		
Contrasts				
session [pre]	−1			
session [post]	1			
group [AC]	−1			
group [ST]	1			

Interactions are indicated by the symbol “:”. GLMM configuration: score ~ 1 + session * group + pta_z + age_z + (1 | subject).

Bold values indicates statistical significant *P* values (*P* < 0.05).

### Significant association between objective and subjective hearing

**Fig. 7:**
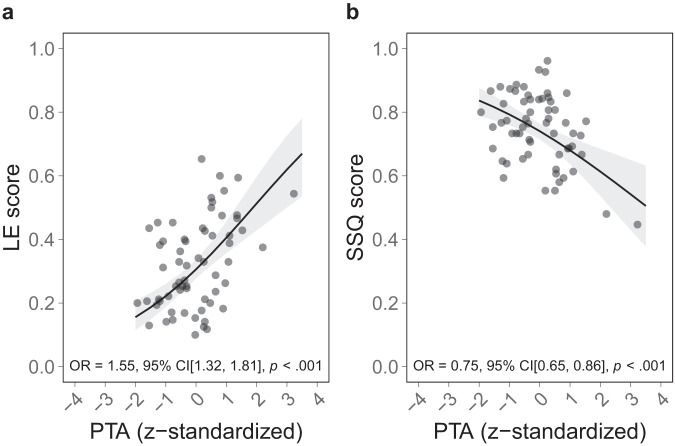
Relationship between objective and subjective hearing. Model predictions for the significant relationship between objectively measured hearing (PTA) and subjective hearing from the generalized linear mixed-effects models. PTA significantly modulates subjective hearing, both in (**a**) LE and (**b**) SSQ. Shaded areas depict 95% confidence intervals. Each dot represents an individual participant. LE Listening Effort Questionnaire, SSQ Speech, Spatial and Hearing Qualities.

Corroborating the results from a previous study^8^, the models showed that PTA was a significant predictor, both for listening effort (OR = 1.55, 95% CI[1.32, 1.81], *p* < 0.001) and the speech, spatial, and hearing qualities (OR = 0.75, 95% CI[0.65, 0.86], *p* < 0.001) (Tables 3, 4 and Fig. 7). A higher PTA (i.e., a higher level of hearing loss) was associated with increased self-perceived listening effort and lower self-perceived hearing qualities in daily life.

## Discussion

Hearing loss is the most prevalent sensory impairment in older adults and its prevalence will continue to grow with the aging of western societies^1^. Although hearing aids have made tremendous technological advances in recent years, they do not provide relief in every clinical case of hearing loss^3^. The use of other strategies such as speechreading trainings could provide a valuable complement in an auditory rehabilitation plan. In the current study we investigated whether a self-guided speechreading training improves audiovisual speech perception in noise in a sample of middle-aged and older adults. For this purpose, we compared data from a sample of 32 participants completing three weeks of computer-based speechreading training and an active control with 30 participants learning a foreign language. Pre- and post-training, participants completed a speech-in-noise comprehension task using sentences mimicking everyday communication situations and answered questions about their subjectively perceived hearing abilities.

Using generalized linear mixed-effects models our analyses revealed a significant improvement in speech comprehension in noise in the speechreading training group for the AV condition only. This is an important finding as it underscores the potential of an inexpensive support tool for adults with communication problems. The relevance of treating these problems that arise in older age is underscored by the proposed link between hearing loss and the increased risk for dementia^7^. Any form of intervention that counteracts this risk factor is a relevant support for healthy aging.

Our finding is in line with previous studies showing positive effects of speechreading trainings^12,14,15^. Embedding our results in the existing literature is however difficult as most of these studies focused on training restricted to visual stimuli (i.e., no audio stream was presented during the training phase) and measured performance in isolated viseme consonant recognition^12,14,15^. Although some studies did use speech perception in noise as outcome measure^13,15^, methodological factors make direct comparisons with our results difficult. Apart from the small sample sizes the sentence material used to measure speech perception by those previous studies was either partially presented repeatedly to the same subjects^13^ or the entire test set was used at each measurement time point^15^ possibly biasing the results. Furthermore, the tasks used required participants to repeat key words from the sentences heard. Our task on the other hand required participants to grasp the gist of the sentence—a central factor of successful everyday communication. It should be mentioned that no other variables modulated the learning rate. However, future research should focus on possible covariates that have an impact on learning success, as this may serve as important information for clinicians to make an informed decision about the use of such a speechreading training with their patients.

In contrast to the performance in speech comprehension, the training had no significant effect on subjective hearing. Although only conjectural, this could underline the robustness of the effects found. In other words, it could be a possible indication that expectancy effects in the ST group might not have had an impact on the outcome measured.

The ultimate goal of intervention studies is to causally attribute the outcome of interest to the treatment with randomized controlled trials being considered the gold standard^19^. In contradiction to these guidelines, we decided on using a between-group design at the expense of the highest level of evidence possible. As we wanted to maximize participants’ motivation to adhere to the prescribed training intensity and as they should solve the training tasks to the best of their ability, we refrained from using a randomized group assignment. Another possibility for training studies are cross-over trials where the participant receives all treatments—in the current study being language and speechreading training—and therefore serves as his or her own control^20^. As with randomized controlled trials we decided against this kind of study design as we wanted to prevent participants from having to do a training they did not want to do and therefore prevent differences in motivation to bias the results. Furthermore, as solid knowledge is lacking in this field of research, it is unclear how long the “washout period” between the consecutive treatments should last to avoid possible carry-over effects. Although the between-group design can be considered a limitation of the current study, it was a deliberate decision after carefully regarding other possibilities.

The determination of the individualized signal-to-noise ratio (SNR) might be considered a further limitation. As the individual 50% speech perception threshold in noise was determined using an adaptive matrix sentence test (i.e., the Oldenburg sentence test (OLSA)^21^) and applied to our naturalistic set of stimuli, a direct transfer of the SNR determined therein was not appropriate (see section *Stimulus preprocessing*). Since the OLSA uses sentences with no predictability between words that are embedded in speech-shaped noise, a direct transfer of the 50%-threshold determined therein to our stimuli was not possible. Since our stimuli contain contextual information and the participants can draw on their background knowledge due to the proximity to everyday life, embedding them in speech-shaped noise at the SNR determined in the OLSA would have resulted in a significantly higher performance in A-only. In a pilot study we therefore tested noise with different numbers of background talkers and found 48 talkers resulting in performance closest to 50%. However, as piloting was done on a sample of young normal hearing adults (*N* = 10; *M*_Age_ = 26.90, *SD*_Age_ = 3.60, Range_Age_ = 20–33; PTA < 25 dB HL) we did not consider the higher susceptibility to a variable masker (as it still is the case with 48 background talkers) and the considerable interindividual variability in older adults. As depicted in Fig. 2, performance was widely spread around the mean, which is below 50%. This could also be seen in a previous study that employed a matrix sentence test to determine the speech perception threshold of interest and applied the SNR to another set of stimuli^8^. It is undeniable that in an experimental setting where A-only and AV conditions are considered, the SNR must be determined individually for A-only to prevent ceiling effects in AV—and even more so when potential improvements in AV are to be measured. When using naturalistic stimuli such as in the present study, it might be preferable to determine the SNR with the same kind of stimuli. Due to the acoustic variability of such stimuli, it is however expectable that significantly more trials are needed to determine a certain performance threshold adaptively. The relevance of equalizing perceptual performance between participants is further underscored by our data with participants improving from pre- to post-training (i.e., learners) having a lower baseline score. As mentioned above (see section *Speechreading training improves audiovisual speech perception in noise*) baseline and change scores are inherently correlated in clinical trials^18^. Disregarding baseline imbalances between two study groups may lead to false-positive results where a positive effect is falsely attributed to the treatment.

Another point that can be considered a limitation is the potential lexical overlap between the material in the speechreading training and the sentences used in our experiment. Since there were lessons that specifically used sentences as training material, participants could have just learned to identify individual words which might have resulted in an overestimation of the training effect. We therefore ran a follow-up analysis to compare the test and training set with results suggesting a small lexical overlap. We then correlated the amount of time that was spent during training in sessions where sentences were specifically trained with the learning effects in the audiovisual condition which showed no significant effect (*r* = 0.04, *p* = 0.837). We interpret these findings as support for the assumption that potential lexical overlaps between test and training set were not a (relevant) confounding factor for the observed training effect.

The variability of naturalistic stimuli and the importance of appropriate statistical modeling thereof is underscored by the random effects reported in Table 2. The between-item variance explains a large portion of the total variance. Not accounting for such clustering in a given data set may lead to unacceptably high Type I error rates with possibly unreliable results^22,23^ and statistical inferences that are not generalizable to a broader set of stimuli^24^. We argue that by accounting for the sampling of participants and items by means of introducing these variance components in the random effects structure, our results are likely to generalize beyond the boundaries of the present study^24^. Furthermore, the naturalistic stimuli and the task type used in the current study are arguably a better representation of communication settings one encounters in everyday life, with comprehension scores determined therein presumably approximating real-life comprehension more accurately.

In summary, the current study showed the beneficial effects of a computer-based, self-guided speechreading training for speech comprehension in noise in a sample of normal to moderately hearing-impaired middle-aged and older adults. Since hearing aids do not mitigate communication problems in every clinical case^3^, computer-based speechreading trainings offer a valuable complement that is cheap, easily applicable and incorporable into daily life. Furthermore, we used a novel naturalistic approach with participants being required to answer questions that relate to everyday life. This question format arguably allows for a better transfer to comprehension performance in everyday life which the tasks used so far do not allow.

## Methods

### Participants

A sample of 63 right-handed German speaking adults participated in the present study. Participants were recruited through two different study announcements containing information about a study investigating the effect of either a speechreading or a language training on speech comprehension in noise. Participants could therefore decide for themselves whether they wanted to join the speechreading or the language learning group. Since one participant only trained on one day, he was excluded from all further analyses. The final sample consisted of 62 adults, with 32 completing the speechreading (*M*_Age_ = 67.53 years, *SD*_Age_ = 7.82 years, Range_Age_ = 50–83 years, 16 female) and 30 completing the language training (*M*_Age_ = 71.70 years, *SD*_Age_ = 7.42 years, Range_Age_ = 47–82 years, 16 female). Demographic information and summary statistics of hearing and cognitive abilities are listed in Table 1 and depicted in Fig. 1. Participants had no history of neurological or psychiatric disorders and showed no sign of cognitive impairment (Montreal Cognitive Assessment ≥26^25^). Furthermore, participants reported no speech or language deficits (e.g., dyslexia) and professional musicians were excluded. Participants’ hearing was assessed using a MAICO ST 20 audiometer (MAICO Diagnostics, Berlin, Germany) and HDA 280 headphones (Sennheiser, Wedemark, Germany). PTAs were calculated by averaging thresholds over the octave frequencies from 0.5 to 8 kHz (*M*_PTA_ = 37.39, *SD*_PTA_ = 14.13, Range_PTA_ = 10–83). Seven participants in the ST group and eight participants in the AC group were hearing aid users who wore their hearing aid throughout the duration of the study. Visual acuity was assessed using the Pelli-Robson Contrast Sensitivity test^26^ and the Snellen test^27^ to ensure that the instructions on the computer could be read and the stimuli could clearly be seen. All participants gave their written informed consent and received monetary compensation for participation. The study was conducted in accordance with the Declaration of Helsinki and approved by the local ethics committee (University of Zurich Ethics Commission, approval number 20.12.20).

### Cognitive tasks

#### Visual working memory

At pre-training, participants completed two tasks measuring cognitive functions appearing to be predictive for lipreading abilities: visual working memory and processing speed^17^. Visual working memory was measured using a computerized version of the Corsi Block-Tapping task^28^. The task consists of nine randomly arranged squares presented on screen with squares lighting up in random sequences participants must memorize and reproduce in reverse order. After every two trials, the sequence length is increased by another square. The task continues until participants make an error in both sequences of a list length. The task starts with sequences of two blocks and goes up to nine blocks. For each participant, the total score was calculated (i.e., the product of the span length and the number of correct trials; possible scores [0, 144]) as it is regarded as a more reliable measure than a simple block span score (i.e., the number of blocks of the longest sequence correctly remembered; possible scores [0, 9])^29^.

#### Processing speed

Processing speed was measured using the Digit-Symbol-Coding test, a Paper-and-Pencil subtest from the Wechsler Adult Intelligence Scale-III^30^. In this test, participants are required to complement numbers with certain symbols according to a key located on top of the page. After 90 s the number of correct symbols is counted. For statistical analyses, raw scores were transformed to scaled scores with a mean of 10 and a standard deviation of 3 points.

### Subjective hearing

At pre- and post-training participants completed two questionnaires on subjective hearing in everyday life: the Listening Effort Questionnaire (LE)^31^ and the German short version of the Speech, Spatial and Qualities of Hearing Scale (SSQ;^32^ German short version^33^). In the LE questionnaire, participants rate their perceived listening effort on a scale from 0 (not exhausting) to 10 (extremely exhausting) for 17 different listening situations. The SSQ German short version consists of 17 questions where participants rate their perceived hearing abilities in different daily-life situations from 0 (none) to 10 (perfect).

### Training procedures

#### Speechreading training

The ST group trained speechreading using the training software by pro audito Schweiz (freely accessible on www.lippenlesen.ch). The training contains eight lessons structured in the same way: 1) an introductory video summarizes the content and objective of the respective lesson, 2) in various flashcards the contents are trained, and 3) in exercises the contents trained are tested. In lesson one, participants are informed about which information is conveyed by the speaker’s face and which is not. In lesson two, the mouth forms of vowels and diphthongs are practised. In lessons three, four, and five, mouth forms of different consonants are trained and tested. The learning content is conveyed by means of mirror exercises and learning videos. Exercises test what has been learned, for example, recognizing syllables in words or differentiating words with minimal syllable differences. In lesson 6, the importance of contextual information is emphasized. In several exercises, participants answer questions in different contexts (i.e., at the cash desk, in the restaurant, and at the post office). In lesson 7, numbers, days of the week, months, and names are trained. In several exercises, participants must recognize for example dates, amounts of money, and more. In lesson 8, speech perception in noise is trained and tested where they need to follow a story and answer questions about the story.

#### Language training

The AC learned Spanish, English, or French through the language learning software Duolingo. Participants were free to choose which language they wanted to learn to maximize motivation and adherence. Furthermore, the exercises to be worked on could also be freely chosen. Both the ST and the AC were instructed to practise for 20 min on each of five days per week over a three-week period with free choice of the timing of a session. During the training period, participants in both groups kept a learning diary in which they recorded the exercises solved and the time spent per learning unit. As Duolingo only provides the number of experience points with detailed logfiles not being available, the AC was asked to complete 50 experience points per learning day which takes around 20 min to complete. From the ST group, on the other hand, logfiles were available and could be directly compared with the training times reported in the learning diaries. Although the experience points represent an approximation of the training time, it cannot be excluded that the reported training times of the AC are not as accurate as the exact logfile data of the ST.

### Stimuli and experimental paradigm

**Fig. 8:**
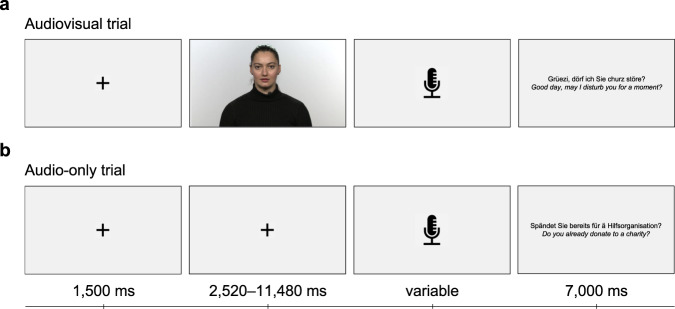
Speech-in-noise comprehension task paradigm. Participants were presented with (**a**) audiovisual and (**b**) audio-only trials. Timeline for a trial is shown at the bottom of the plot. The speaker gave her consent to publish her photograph.

Video recordings of a female speaker were used as stimulus material. Videos showed the speaker’s head and shoulder in front of a plain, non-distracting background. The speaker’s face pointed directly into the camera and was well lit so that her face was fully visible and not in shadows. The speech material consisted of questions depicting everyday situations that were spoken in Swiss German (*M*_SentenceLength_ = 5641 ms, *SD*_SentenceLength_ = 1771 ms, Range_SentenceLength_ = 2520–11,480 ms) (Supplementary Table 1). Participants were instructed to give an answer that clearly indicated whether they understood the question or not (yes/no answers were prohibited). Questions were presented in blocks of five sentences each. Each block formed a hypothetical scenario one could encounter in everyday life, with the questions contained within revolving around the same topic. The rationale was that this comes closest to a realistic communication setting where the listener not only perceives the acoustic signal, but can draw on other cues (e.g., contextual information). After the question was asked, the picture of a microphone appeared on the screen, signaling to the participant that an answer should be given. After an answer was given, the previously asked question was displayed on the screen for seven seconds. This would ensure that the participant could grasp the context, even if he or she had not understood the previously asked question, and therefore not biasing the answer to the next question. Trials were either presented in blocks with (AV) or without the video (A-only) of the speaker. Participants completed six AV and six A-only blocks in each session (i.e., 60 trials in each session). The presentation order of blocks and condition was randomized. The experiment started with a training block where five AV stimuli were presented. The experimental paradigm is outlined in Fig. 8.

### Stimulus preprocessing

To create the stimuli, silent periods with a duration of ≥100 ms were cut out before normalizing the root mean square to 70 dB. Afterwards, silent periods were reinserted. This procedure was required as speech segments in sentences containing longer pauses would have been louder after normalization and embedding in background noise. Since the speaker memorized each sentence before saying it directly into the camera and was instructed to speak naturally, the recordings contained more pauses than if she had just read the sentences aloud. The background noise was created by overlaying 48 randomly selected recordings of the same speaker while ensuring for each trial that the noise mixture did not contain the respective target sentence. It should be noted that the number of background talkers was not chosen at random. A primary goal was to individualize the SNR so that performance lies around 50% in A-only to prevent possible ceiling effects in AV at pre-training which would have made it impossible to measure potential training effects. To find the individual 50% speech perception threshold in noise, we used an adaptive German matrix sentence test (i.e., OLSA). Since the OLSA uses sentences with no predictability between words that are embedded in speech-shaped noise, a direct transfer of the 50%-threshold determined therein to our stimuli was not possible. Since our stimuli contain contextual information and the participants can draw on their background knowledge due to the proximity to everyday life, embedding them in speech-shaped noise at the same SNR would have resulted in a significantly higher performance in A-only. This was the reason why we needed a more difficult setting so that performance in A-only converged to around 50%. In a pilot study, young normal hearing adults (*N* = 10; *M*_Age_ = 26.90, *SD*_Age_ = 3.60, Range_Age_ = 20–33; PTA < 25 dB HL) completed the OLSA. The individual 50% speech perception threshold (i.e., SNR) was then used on our set of stimuli. In a next step we tested different numbers of background talkers (i.e., 6, 12, 24 and 48 talkers) with performance in 48-talker background noise being closest to the targeted 50%. Stimulus preprocessing was done in Praat^34^ (version 6.1.40) and MATLAB^35^ (version R2021b) using custom-made scripts. Video recordings had a resolution of 1,920 ×1,080 pixels with 25 frames per second while audio was recorded with a sampling rate of 44.1 kHz and resolution of 16 bits. Stimuli were controlled via sound card (RME Babyface Pro, RME Audio, Haimhausen, Germany) and presented through a Genelec 8030B Studio Monitor loudspeaker positioned at 0° azimuth with linear frequency response (Genelec, Iisalmi, Finland) at an intensity of 70 dB(A). Sound level calibration was done using an NTi XL2 sound level meter (NTi Audio, Schaan, Liechtenstein). Participants’ responses were recorded using an MKE 600 microphone (Sennheiser, Wedemark, Germany) placed at 60° azimuth ensuring a clear view on the screen.

### Statistical analyses

#### Group comparisons

In a first step, sample characteristics of the speechreading training and the active control group were compared. This was particularly important as we wanted to rule out the possibility of variables of non-primary interest being responsible for potential training effects. Using Welch’s two sample *t*-tests we compared the two groups in terms of PTA, age, training intensity as well as visual working memory and processing speed. Welch’s *t*-test was used as it is generally recommended when comparing groups of unequal sizes and/or the assumption of variance homogeneity is not met^36^. All statistical tests were conducted in R^37^ (version 4.0.3).

#### Predictors of visual enhancement

In a next step we investigated potential predictors of VE at pre-training with VE being defined as the difference between A-only and AV^10^. VE was measured by means of a normalized difference score according to the following formula (1) (e.g.,^10^)

1 $$VE=(AV-A{\hbox{-}}only)/(1-A{\hbox{-}}only)$$

The normalized difference score accounts for baseline differences by considering the potential for improvement in A-only as opposed to a simple difference score (i.e., *AV* − *A-only*)^10^. Using multiple regression analysis, the relationship between VE and several variables (i.e., PTA, age, visual working memory, and processing speed) was investigated. All predictors were z-standardized.

#### Training effects on speech-in-noise comprehension

The primary goal of the present study was to investigate the effect of speechreading training on performance in a speech-in-noise comprehension task. Since we used an open response format, finding an unbiased method for scoring was key. For this purpose, two blinded independent raters scored participants’ answers (right or wrong) with ambiguous answers being excluded from subsequent analyses (3.18%). Cohen’s kappa, a measure of interrater reliability, indicated an almost perfect agreement between the two raters (*κ* = 0.91, 95% CI[0.90, 0.92])^38^, suggesting that most of the responses were clear. Subsequently, trials where the two raters agreed in their rating were extracted and analyzed. Given the dependencies among data points (i.e., each participant completed several trials in two conditions at two different time points and each item was presented in each condition, session, and group) it was deemed appropriate to fit a GLMM specified as binomial model with a logistic link function. The binary response measure was coded as 1 and 0 based on the participants’ responses (1 = correct; 0 = incorrect). In a first step, the model with the maximal random effects structure justified by the design was estimated^23^ using the *lme4* package^39^. The model included fixed effects of condition (categorical variable with two levels: A-only, AV), session (categorical variable with two levels: pre-training, post-training), and group (categorical variable with two levels: AC, ST), the three-way interaction between condition, session, and group, as well as the covariate age (continuous variable: in years) which was z-standardized. As for the random effects, the model included by-subject and by-item random intercepts (i.e., crossed random effects^22^). Following the general rule of fitting a random slope for each focal within-unit predictor, the model contained by-subject random slopes for condition, session, their interaction, as well as by-item random slopes for condition, session, group, and their interaction. The random effects structure was further simplified by iteratively removing random effect terms until a non-singular fit was achieved^23^. We refrained from using a stepwise approach to determine the fixed effects specified in the fitted model (i.e., by means of comparing an encompassing model with a reduced model that omitted a fixed effect of interest). Rather, we wanted to specify the potential training effect—that is whether the experimental group improved from pre- to post-training in AV—as a regression model depicted as three-way interaction between condition, session, and group. The default contrast coding scheme (i.e., *treatment coding*) was changed to sum-coding (i.e., *effect coding*) where the lower-level effects (i.e., main effects) are estimated at the level of the grand mean and interpreted accordingly^40^. In addition to the fixed effects, variance estimates of the random effects are also reported. Model assumptions were checked using the *performance* package^41^ and model predictions were plotted using the *ggeffects* package^42^. The maximal model was defined as specified in formula (2) with random intercepts and slopes being indicated by *S*_0*s*_ and *S*_x ≥ 1*s*_ for subject as well as *I*_0*i*_ and *I*_x≥1*i*_ for item.

2 $$respons{e}_{si}={\beta }_{0}+{S}_{0s}+{I}_{0i}+({\beta }_{1}+{S}_{1s}+{I}_{1i})condition+({\beta }_{2}+{S}_{2s}+{I}_{2i})session+({\beta }_{3}+{I}_{3i})group+{\beta }_{4}age\_z+({\beta }_{5}+{S}_{5s}+{I}_{5i})condition\ast session+({\beta }_{6}+{I}_{6i})condition\ast group+({\beta }_{7}+{I}_{7i})session\ast group+({\beta }_{8}+{I}_{8i})condition\ast session\ast group+{e}_{si}$$

#### Training effects on subjective hearing

In a further step, we investigated potential training effects on subjective hearing. For this purpose, we obtained average scores for both questionnaires (i.e., LE and SSQ) in each session. To account for the restricted range in the two outcome measures (i.e., [0, 10]), we fitted two GLMMs (i.e., one for LE and one for SSQ) with a beta distribution and a logistic link function using the *glmmTMB* package^43^. As the beta distribution has a range restriction of (0, 1), we divided the questionnaire scores by 10 before fitting the models. As the transformed data did not include 0 or 1, no further transformation was necessary. The models included fixed effects of session and group, the two-way interaction between session and group, as well as the covariate age. As we were interested in exploring the relationship between subjective and objective hearing, we included PTA as further covariate (continuous variable: in dB HL). As there was only one score per participant for each session, estimating random slopes was not possible. To account for the dependencies in the data, we fitted a by-subject random intercept. As above, we refrained from doing stepwise model selection and rather tested specifically whether training effects were present by specifying the two-way interaction between session and group. Orthogonal contrasts were again used as factor coding. The model is described in formula (3) with the by-subject random intercept being indicated by *S*_0*s*_.

3 $$respons{e}_{si}={\beta }_{0}+{S}_{0s}+{I}_{0i}+({\beta }_{1}+{S}_{1s}+{I}_{1i})condition+({\beta }_{2}+{S}_{2s}+{I}_{2i})session+({\beta }_{3}+{I}_{3i})group+{\beta }_{4}age\_z+({\beta }_{5}+{S}_{5s}+{I}_{5i})condition\ast session+({b}_{6}+{I}_{6i})condition\ast group+({\beta }_{7}+{I}_{7i})session\ast group+({\beta }_{8}+{I}_{8i})condition\ast session\ast group+{e}_{si}$$

### Reporting summary

Further information on research design is available in the Nature Research Reporting Summary linked to this article.

### Supplementary information

Supplemental material

Reporting Summary

## Data Availability

The complete data as well as the audiovisual stimuli used in this study are publicly available in the study’s Open Science Framework repository (https://osf.io/35tzm/).

## Code availability

The analysis code to reproduce reported findings are publicly available in the study’s Open Science Framework repository (https://osf.io/35tzm/).
