# Supplementary material for: Improvements in naturalistic speech-in-noise comprehension in middle-aged and older adults after 3 weeks of computer-based speechreading training
Source: NPJ Sci Learn. 2023 Sep 4;8:32. doi: 10.1038/s41539-023-00179-6 (PMC10477252; doi:10.1038/s41539-023-00179-6)
Supplement: Supplementary file 1 — Supplemental material [file 41539_2023_179_MOESM1_ESM.pdf]

Supplementary Information

for

IMPROVEMENTS IN NATURALISTIC SPEECH-IN-NOISE COMPREHENSION IN MIDDLE-AGED AND OLDER ADULTS AFTER THREE WEEKS OF COMPUTER-BASED SPEECHREADING TRAINING

Raffael Schmitt[1,2,3*],

Martin Meyer[4,5,6],

Nathalie Giroud[1,2,3,7]

[1]Department of Computational Linguistics, University of Zurich, Zurich, Switzerland
[2]International Max Planck Research School on the Life Course: Evolutionary and Ontogenetic Dynamics (LIFE)
[3]Language & Medicine Centre Zurich, Competence Centre of Medical Faculty and Faculty of Arts and Sciences, University of Zurich, Zurich, Switzerland
[4]Department of Comparative Language Science, University of Zurich, Zurich, Switzerland
[5]Center for the Interdisciplinary Study of Language Evolution (ISLE), University of Zurich, Zurich, Switzerland
[6]Cognitive Psychology Unit, Alpen-Adria University, Klagenfurt, Austria
[7]Neuroscience Center Zurich, University of Zurich and ETH Zurich, Zurich, Switzerland

***Corresponding author:**
Raffael Schmitt, M.Sc.
Department of Computational Linguistics
University of Zurich
Andreasstrasse 15
8050 Zurich
raffael.schmitt@uzh.ch

This document includes

Supplementary Figures 1, 2, and supplementary tables 1, 2, 3

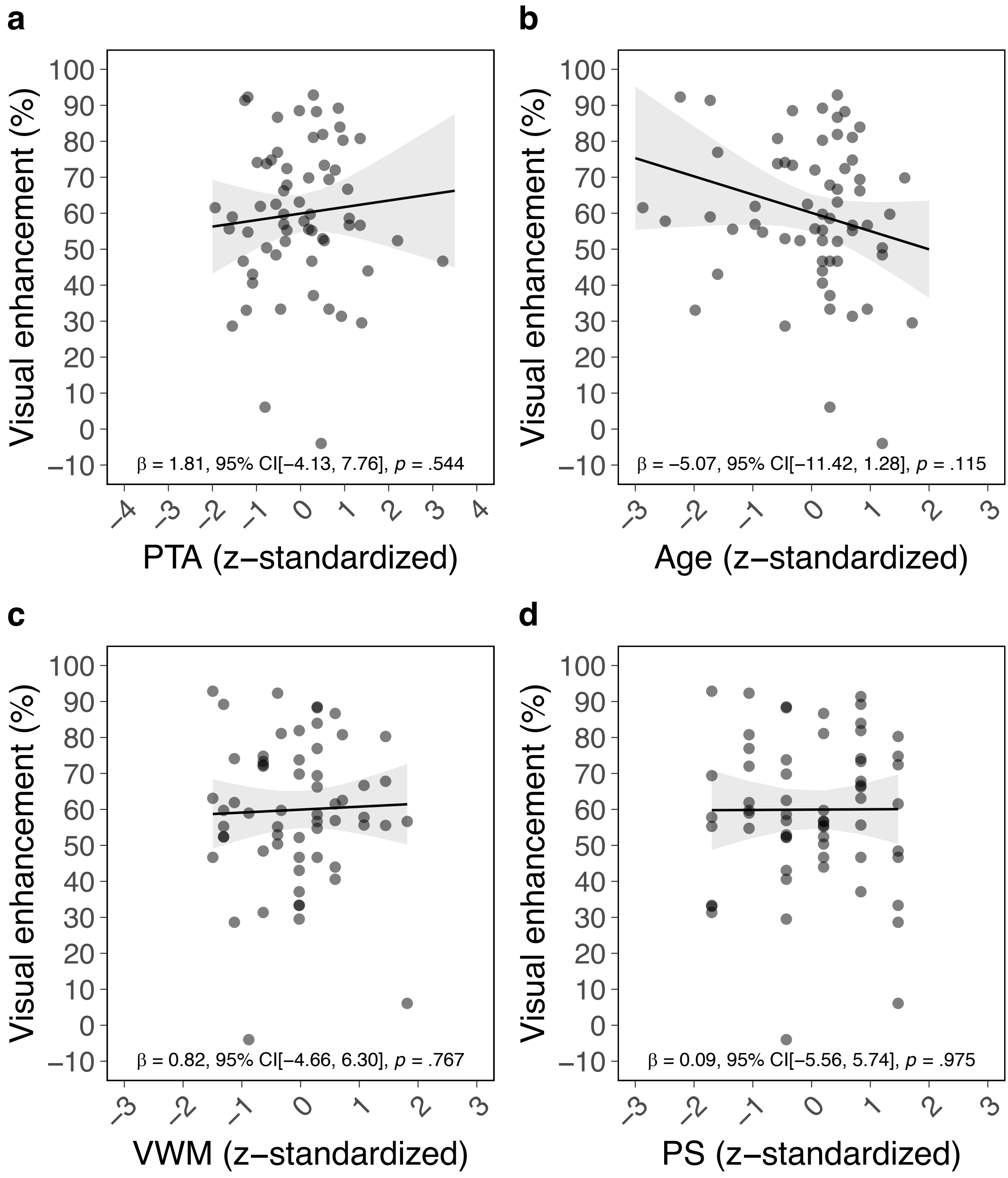

Supplementary Figure 1: Possible predictors of visual enhancement. Model predictions for the non-significant effects of **a** PTA, **b** age, **c** visual working memory, and **d** processing speed on visual enhancement at pre-training from the multiple regression analysis. Shaded areas depict 95% confidence intervals. Each dot represents an individual participant. VWM, visual working memory; PS, processing speed; β, standardized beta coefficient.

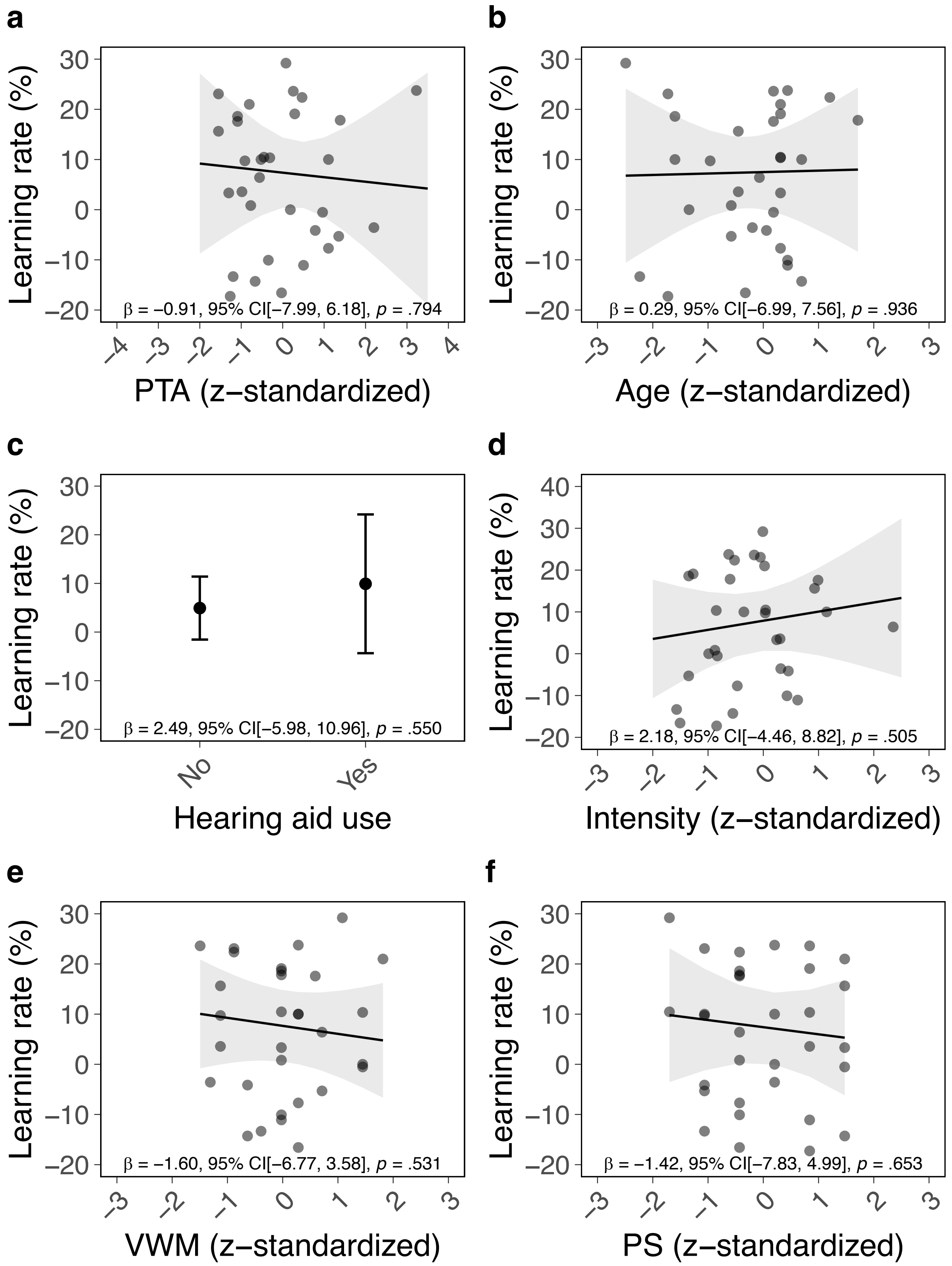

Supplementary Figure 2: Possible predictors of learning rate. Model predictions for the non-significant effects of **a** PTA, **b** age, **c** hearing aid use, **d** training intensity, **e** visual working memory, and **f** processing speed on learning rate in the speechreading training group. Shaded areas (**a, b, d, e, and f**) and bars (**c**) depict 95% confidence intervals. Each dot represents an

individual participant. VWM, visual working memory; PS, processing speed; β, standardized beta coefficient.

Supplementary Table 1. Stimuli set with English translation in italics.

1_1	Grüezi, dörf ich Sie churz störe?<br>*Good day, may I disturb you for a moment*
1_2	Sind Sie vo Züri?<br>*Are you from Zurich?*
1_3	Ich bin uf de Suechi nachere Migros. Chönnd Sie mier da wiiterhelfe?<br>*I am looking for a Migros (largest grocery shop chain in Switzerland). Can you help me there?*
1_4	Ou, ich bruuch no Bargeld. Wüssed Sie wo die nächsti UBS-Filiale isch?<br>*I need cash also. Do you know where the nearest ATM is?*
1_5	Chönnted Sie mir villicht grad no säge, wie ich zum HB chume?<br>*Could you also tell me how to get to the main station?*

2_1	Schön Sie wieder emal z'gseh. Gahts Ihne guet?<br>*Nice to see you again. How are you?*
2_2	Was mached denn Sie da vor dä Uni Züri?<br>*What are you doing in front of the University of Zurich?*
2_3	Isch das die ersti Studie, wo sie teilnemed?<br>*Is this the first study you have participated in?*
2_4	Wie sind Sie denn uf die Studie cho?<br>*How did you learn about this study?*
2_5	Uf äm Online Marktplatz vo dä Uni Züri gits au immer wieder Inserat. Känned Sie dä?<br>*There are always ads on the online marketplace of the University of Zurich. Do you know it?*

3_1	Guete Morge. Händ Sie en Moment Ziit?<br>*Good morning, do you have a moment?*

3_2	Mier sind vo dä Uni Bern und mached ä chlini Umfrag. Interessieret Sie sich für Politik?<br>*We are from the University of Berne and are doing a small survey. Are you interested in politics?*
3_3	Wenn Sie ageh müssted, wie hüfig Sie bi Abstimmige mitmached, wäri das «immer», «fascht immer», «selte» oder «nie»?<br>*If you had to indicate how often you vote, would it be "always", "almost always", "rarely" or "never"?*
3_4	Woned Sie inere Stadt, oder inere ehner chlinere Gmeind?<br>*Do you live in a city or in a rather smaller town?*
3_5	Chönnted Sie mir ächt no säge, wie alt Sie sind.<br>*Could you tell me how old you are.*

4_1	Händ Sie gseh, dass es i dä Migros es paar gueti Aktione hät?<br>*Have you seen that Migros has some good discounts.*
4_2	D'Aubergine sind im Momänt abegsetzt. Händ sie gwüsst, dass es Aubergine us dä Schwiiz git?<br>*The eggplants are cheaper at the moment. Did you know that there are eggplants from Switzerland?*
4_3	Ich chaufe die nume no i dä Aktion. Lueged Sie au ä chli druf, was grad abegsetzt isch?<br>*I only buy them when they are discounted. Do you also pay attention to what is dis-counted?*
4_4	Ich lise jedi Wuche dä Brügglibuur. Händ Sie dä au abonniert?<br>*I read the "Brügglibuur" (grocery magazine) every week. Have you also subscribed to it.*
4_5	Und d'Coop-Ziitig? Läsed Sie die?<br>*And the Coop-Magazine (another grocery magazine). Do you read it?*

5_1	Guete Morge. Wänd Sie lieber es Gipfeli oder es Brötli?<br>*Good morning. Would you prefer a croissant or a roll?*
5_2	Wänd Sie grad no en Kaffi dezue?<br>*Would you like a coffee with that?*
5_3	Hüt Morge hämmer grad no frischi Zöpfli gmacht. Wänd Sie eine?<br>*This morning we just made fresh bernese braids. Would you like one?*

5_4	Das macht en Franke Nünzg. Händ sies gnau?<br>*That's 1.90.-. do you have it exactly?*
5_5	Chönnted Sie ächt miteme Foifliber zalä? Ich gsehne grad, das mer kei meh händ.<br>*Can you pay with the fiver? I just saw that we don't have any more.*

6_1	Danke fürs Warte. Ich bi grad in Stau cho mitem Velo. Bisch du mit dä ÖV cho?<br>*Thanks for waiting. I just got stuck in traffic on my bike. Did you come by public transport?*
6_2	Ich fahre sit dere Corona Gschicht viel hüfiger Velo. Du au?<br>*I've been cycling a lot more since the pandemic. You too?*
6_3	Ich hamer überleit ä sonens Elektrovelo uszprobiere. Bisch mal mit so eim gfahre?<br>*I've been thinking about trying out an electric bike. Have you ever ridden one?*
6_4	Ich find die Velowäg in Züri chönnteds ä chli usbaue. Was haltisch du vo dene?<br>*I think the cycle paths in Zurich could be expanded a little. What do you think of them?*
6_5	Findsches nöd au gföhrlich wenn d Velofahrer so a dä Fuessgänger verbiirased?<br>*Don't you think it's dangerous when cyclists speed past pedestrians?*

7_1	Grüezi, mier mached ä chlini Umfrag. Nutzed Sie Google?<br>*Hello, we are doing a small survey. Do you use google?*
7_2	Nutzed Sie euses Charteapp «Google Maps»?<br>*Do you use our map app google maps?*
7_3	Känned Sie d'Funktion «Google Streetview»?<br>*Do you know the google streetview function?*
7_4	Mit Google Streetview chamer virtuell uf dä Strass umespaziere. Was haltet Sie devo, wemmer das au innerhalb vo bestimmte Gebäude chönnt?<br>*With google streetview you can virtually walk around on the street. What would you think if you could also do that inside certain buildings?*
7_5	Mier wennd ermögliche, dasmer mitem Computer i historische Gebäude i schwiizer Städt virtuell umelaufe chan. Was haltet Sie vo dere Idee?<br>*We want to make it possible to virtually walk around historic buildings in swiss cities with a computer. What do you think of this idea?*

8_1	Du etz hani grad vonere Studie glese, das Mensche mit Hund zfriedener sind. Häsch du Än Hund?<br>*I just read about a study that people are happier with dogs. Do you have a dog?*
8_2	Häsch du suschtigi Huustier?<br>*Do you have any pets?*
8_3	Ich überlegmer scho lang es Büsi zueztue. Häsch du gern Chatze?<br>*I have been thinking about getting a cat for a long time. Do you like cats?*
8_4	Än Hund hani ja scho lang. Meinsch de verstaht sich guet mitere Chatz?<br>*I have had a dog for a long time. Do you think he gets along well with a cat?*
8_5	Wennd wähle chönntsch, was würsch füres Huustier welle?<br>*If you could choose, what would you choose for a pet?*

9_1	Guete Morge, händ Sie en Moment Ziit?<br>*Good morning, do you have a moment?*
9_2	Spändet Sie bereits für ä Hilfsorganisation?<br>*Do you already donate to a charity?*
9_3	Mir sammlet im Moment Spände für dä Schutz vom ostafrikanischä Berggorilla. Känned Sie dä?<br>*We are currently collecting donations for the protection of the East African mountain gorilla. Do you know them?*
9_4	Händ Sie gwüsst, dass die Art vom Ussterbe bedroht isch?<br>*Did you know that this species is on the verge of extinction?*
9_5	Wäred Sie bereit än Spändebiitrag z’zahle?<br>*Would you be willing to pay a donation?*

10_1	Hüt Abig lauft Sportler des Jahres im Fernseh. Luegsch das au?<br>*Tonight sportsman of the year is on TV. Are you watching it too?*

10_2	Letschmal het sonen Velofahrer gunne. Interessiersch du dich für de Radsport?<br>*Last time, a cyclist won. Are you interested in cycling?*
10_3	Ja, ich mich nöd eso. Und Wintersport?<br>*Yes, I do not so. And winter sports?*
10_4	Aber am Federer luegi gern zue. Gfallt dier Tennis?<br>*But Federer I like to watch. Do you like tennis?*
10_5	Weisch, ich ha früener emal gspillt. Machsch du eigentlich Sport?<br>*You know, I used to play. Do you actually do sports?*

11_1	Entschuldiged Sie d'Störig, aber wüssed Sie, ob mer am Billetschalter au mit Charte zahle chan?<br>*Sorry to bother you, but do you know if you can pay by card at the ticket office?*
11_2	Es isch scho no praktisch, das mer jetzt mit dä Chartä chan kontaktlos zahle. Mached Sie das au?<br>*It is convenient that you can now pay with card contactless. Do you do that too?*
11_3	Ich bin froh, das i äs Halbtax han bi denä Priisä. Händ Sie au es Halbtax?<br>*I'm glad I have a discounted subscription at these prices. Do you have that too?*
11_4	Letscht Wuchenänd bin i uf Davos mit äme Sparbillet. Händ Sie das au scho mal gnutzt?<br>*Last week I went to Davos with a saver pass. Have you ever used it?*
11_5	Dä Zug het hüt scho wieder Verspötig. Nervt Sie das amel au ä bitz?<br>*The train is late again today. Does that annoy you too sometimes?*

12_1	Guete Morge, mier vo dä Emmi verteiläd hüt Gratismuster. Händ Sie lieber Erdbeer oder Schoggi?<br>*Good morning, we at Emmi are giving away free samples today. Would you prefer straw-berry or chocolate?*
12_2	Ah Vanille hetemer au no. Händ sie Lust d'Vanillemilch z'probierä?<br>*We also have vanilla. Would you like to try the vanilla milk?*
12_3	Wänd Sie en Flyer mitneh zum a eusem Gwünnspiel mitmachä?<br>*Would you like to take a flyer with you to enter our competition?*

12_4	De Hauptpriis isch ä Reis is Bünderland oder is Berner Oberland. Was würed sie näh, wänn Sie gwünned?<br>*The main prize is a trip to the bündnerland or the bernese oberland. What would you take if you won?*
12_5	Das würi au. Sind Sie ufem Weg is Büro?<br>*I would too. Are you on your way to the office?*

13_1	Billetkontrolle! Chönnti echt ihres Billet gseh?<br>*Ticket control! Could I see your ticket?*
13_2	Wo gahts ane?<br>*Where are you going?*
13_3	Händ sie ihres Billet online oder am Schalter kauft?<br>*Did you buy your ticket online or at the ticket office?*
13_4	Händ sie au no es Halbtax?<br>*Do you also have a discounted ticket?*
13_5	Ich gsehne grad, dass sie ä Zone z'viel glöst händ. Isch Ihne das bewusst?<br>*I just see that they have bought one zone too many. Are you aware of that?*

14_1	Grüezi, sie wänd es Billet chaufe? Wo wänd Sie ane?<br>*Hello, you want to buy a ticket? Where are you going?*
14_2	Händ Sie es Halbtax?<br>*Do you have a discount ticket?*
14_3	Die Wuche hämmer usserdem no d'SBB-Tagescharte günstiger im Agebot. Känned Sie die?<br>*This week we also have a special offer on the day ticket. Do you know it?*
14_4	Das macht Nünzäh Franke Vierzg. Händ Sie ä Zwänzgernote?<br>*That makes 19.40.-. Do you have a twenty?*
14_5	Jetzt hani es Durenand gmacht. Wie viel bechömed Sie zrugg?<br>*Now I have made a mess. How much do you get back?*

15_1	Entschuldigung, chönnted Sie mier echt rasch hälfe?<br>*Excuse me, could you help me quickly?*
15_2	Händ Sie es Smartphone?<br>*Do you have a smartphone?*
15_3	Ich ha da es neus Smartphone und chume nöd demit z'Schlag. Wüssed Sie wiemer da de Wäg naluege chan?<br>*I have a new smartphone and I can't cope with it. Do you know how to look up a route?*
15_4	Bruched Sie amel das Charteapp?<br>*Do you sometimes use the map app?*
15_5	Ich ha da amel ä chli Müeh wänn i es neus Grät han. Gaht's Ihne da au ä so?<br>*I have a little trouble when I have a new device. Do you feel the same way?*

16_1	Grüezi, wänd Sie en Kaffi oder en Tee?<br>*Hello, would you like some coffee or tea?*
16_2	Hüt hetemer ä Rüebli-Ingwer-Suppe oder ä Spinat-Lauch-Wähe im Agebot. Was hetted Sie lieber?<br>*Today we have a carrot and ginger soup or a spinach and leek cake on offer. Which would you prefer?*
16_3	Zum Dessert gäbtis es Himbeertiramisu. Wär das öppis für Sie?<br>*For dessert we have a raspberry tiramisu. Would that be something for you?*
16_4	Jetzt hani en Fehler gmacht. Anstatt Triamisu gäbtis ä Orangecreme. Wänd Sie glich es Dessert?<br>*Now I have made a mistake. Instead of tiramisu there is orange cream. Would you still like a dessert?*
16_5	Dörfi Ihne susch no öppis bringe?<br>*Can I choose anything else for you?*

17_1	Hoi, mier händ eus scho so lang nüm gseh. Wie gahts dier?<br>*Hello, we have not seen each other for a long time. How are you?*

17_2	Ich bin grad im Kunsthuus gsi. Häsch die neu Usstellig scho gseh?<br>*I just went to the Kunsthaus (art museum). Have you seen the new exhibition?*
17_3	Sie stelled im Momänt am Gerhard Richter sini Kunst us. Gfallt dier dä?<br>*They are currently exhibiting the art of Gerhard Richter. Do you like his art?*
17_4	Welle Künstler gfallt dier suscht no guet?<br>*Which other artist do you like?*
17_5	Ich bin gspannt, wies neue Kunsthuus wird. Weisch du, wänn de Neubau eröffnet wird?<br>*I am curious to see how the new Kunsthaus will turn out. Do you know when the new building will open?*

18_1	Händ Sie dä Kaffi vo da scho mal probiert?<br>*Have you ever tried the coffee from here?*
18_2	Ich finden nöd ä so guet. Wo gits Ihrere Meinig naa de besti Kaffi?<br>*I don't think it's that good. Where do you think the best coffee is?*
18_3	In Züri gahni gern is Kafi Mandarin. Känned sie das?<br>*In Zurich, I like to go to Café Mandarin. Do you know it?*
18_4	Sie wänds ja jetzt abrisse, wills dä Stadelhofe umbaued. Händ Sie vo däm Umbau ghört?<br>*They want to tear it down now because they're rebuilding the station. Have you heard about the rebuild?*
18_5	Das söll ja de glich Architekt sii wo de Bahnhof Stadelhofe baut hät. Wie heisst de scho wieder?<br>*This is supposed to be the same architect who built the train station. What's his name again?*

19_1	Etz muenider öppis verzelle. Kännsch du de Viktor Giacobbo?<br>*Now I have to tell you something. Do you know Viktor Giacobbo? (Swiss comedian)*
19_2	Ja de Komiker. De isch jetzt gegenüber iizoge. Glaubsch das?<br>*Yes, the comedian. He has now moved in across the street. Do you believe that?*
19_3	Ich kännen nume usem Fernseh. Häsch du amel au Giacobbo/Müller gluegt?<br>*I only know him from TV. Did you also watch Giacobbo/Müller? (comedy show)*

19_4	Ja, die Sändig am Sunntig Abig. Luegsch du au öppe die no Fernseh?<br>*Yes the show on sunday evening. Do you watch TV sometimes?*
19_5	Ich find die redet hützutags oft undütlich. Finsch das au?<br>*I think they often speak unclearly today. Do you think so too?*

20_1	Mei, du mini Pflanze sind vo Mehltau befalle. Häsch das au scho mal gha?<br>*My plants are affected by powdery mildew. Have you also had this before?*
20_2	Weisch villicht, was mer degäge mache chan?<br>*Do you know what to do about it?*
20_3	Häsch du gern Pflanze?<br>*Do you like plants?*
20_4	Ich finds ä schöni Beschäftigung, han aber kein grüene Dume. Wie stahts mit dier?<br>*I find it a nice occupation, but do not have a green thumb. How about you?*
20_5	Letscht Mäntig bini i de Stadtgärtnerei gsi. Die gäbed ganz gueti Tipps. Bisch au scho mal det gsi?<br>*Last Monday I went to the city garden. They give good tips. Have you ever been there?*

21_1	Ich chume mitem Tagi-Chrüzworträtsel eifach nöd vorwärts! Weles isch de tüüfsti See i de Schwiiz?<br>*I just can't get ahead with the crossword puzzle. What is the deepest lake in Switzerland?*
21_2	Ah, de Lago Maggiore. Und weles isch de höchst Berg wo ganz i de Schwiiz staht?<br>*Ah, Lake Maggiore. And which is the highest mountain in Switzerland?*
21_3	Ah genau, das isch ja de Dom. Und wie nännt mer die offene Wasserleitige wo mer im Wallis findet?<br>*Ah, that's right, that's the Dom. And what do you call the open water pipes that you find in the Valais?*
21_4	Suone! So, die letzt Frag: Welli Bündnerspezialität fangt mit «c» aa und hört mit «s» uf?<br>*Suone! So, the last question: which Bündner speciality starts with "c" and ends with "s"?*
21_5	Capuns! Machsch au no öppe die Chrüzworträtsel?<br>*Capuns! Do you do crossword puzzles sometimes?*

22_1	Etz hani grad im K-Tipp glese, dass Kaffi us Guatemala am wenigste Schadstoff beinhaltet. Häsch das gwüsst?<br>*Now I have just read that coffee from Guatemala contains the least pollutants. Did you know that?*
22_2	Lisisch du de K-Tipp?<br>*Do you read the K-Tipp? (consumer magazine)*
22_3	Ich lis de no gern. Oder kennsch susch no öppis, womer ä so gueti Produktbewertige findet?<br>*I enjoy reading it. Or do you know anything else where you can find such good product reviews?*
22_4	Ich ha de K-Tipp jetzt online abonniert und lise de amel uf em Tablet. Lisisch du au Ziitig am Computer?<br>*I have subscribed to K-Tipp online and read it on my tablet. Do you also read newspapers on your computer?*
22_5	Was sind überhaupt no d’Vorteil, d’Ziitige i druckter Version z’läse?<br>*What are the advantages of reading a newspaper in printed form?*

23_1	Du, etz bini grad mitem Elektro-Trottinett da here cho. Bisch scho mal mit dene gfahre?<br>*I just came here on an electric scooter. Have you ever ridden with them?*
23_2	Die stönd ja scho a jedem Egge. Stört dich das?<br>*They're already on every corner. Does that bother you?*
23_3	Ich finds ä chli müesam, wenns zmitzt ufem Weg stönd. Aber susch ischs ja eigentli ä gueti Idee. Oder?<br>*I find it a bit cumbersome when they are in the middle of the path. But otherwise it's a good idea, isn't it?*
23_4	Ou und hesch die chline Elektroautos gseh, womer jetzt au chan miete?<br>*And have you seen the little electric cars that you can rent now?*
23_5	Ich dänke, es wird i Sache Verchehr no einiges passiere. Oder was meinsch?<br>*I think a lot will happen in terms of traffic. Or what do you think?*

24_1	Hesch vo de grösste schwiizer Baustell glese?<br>*Have you read about the biggest swiss construction site?*

24_2	Ja, de Circle am Flughafe Züri. Bisch scho mal dete gsi?<br>*Yes, the Circle at Zurich Airport. Have you ever been there?*
24_3	S'het sogar en Teil vomne Spital det ine. Ah, welle Spital isch das scho wieder gsi?<br>*It even has a part of a hospital. Which hospital was that again?*
24_4	Stimmt, de Unispital. Du und eis vo de grösste Ichaufscenter sötts det ine ha. Gasch du gern i so grossi Ichaufscenter?<br>*Right, the university hospital. And one of the largest shopping centers is supposed to be there. Do you like going to shopping malls that big?*
24_5	Ich verlüür da amel chli de Überblick bi so vielne Läde. Du nöd au?<br>*I sometimes lose track of so many stores. Don't you?*

Supplementary Table 2. Parameter estimates for the effects of learning group (learner vs. non-learner) on performance at pre-training in the speech comprehension task. GLMM configuration: response ~ 1 + condition * session * group + age_z + (1 + condition + session | subject) + (1 + condition + session | item). Treatment contrasts were used as factor coding with "AV" in condition, "pre" in session, and "non-learner" in group as baseline levels. Interactions are indicated by the symbol ":".

Predictors	Odds Ratios	CI	*p*
(Intercept)	7.73	4.74–12.60	**<.001**
condition [A]	0.06	0.04–0.09	**<.001**
session [post]	0.54	0.40–0.73	**<.001**
group [L]	0.54	0.33–0.90	**.018**
age_z	0.92	0.74–1.14	.434
condition [A] : session [post]	1.89	1.26–2.83	**.002**
condition [A] : group [L]	1.84	1.17–2.91	**.008**
session [post] : group [L]	4.00	2.67–5.99	**<.001**
condition [A] : session[post] : group [L]	0.24	0.14–0.41	**<.001**
**Random effects**			
σ^2	3.29		
$\tau_{00\ subject}$	0.72		
$\tau_{00\ item}$	2.83		
$\tau_{11\ subject.condition[A]}$	0.25		

τ_{11} subject.session[post]		0.03
τ_{11} item.condition[A]		0.62
τ_{11} item.session[post]		0.07
**Contrasts**		
condition [A]	1	
condition [AV]	0	
session [pre]	0	
session [post]	1	
group [NL]	0	
group [L]	1	

Supplementary Table 3. Parameter estimates for the effects of group on performance at pre-training in the speech comprehension task. GLMM configuration: response ~ 1 + condition * session * group + age_z + (1 + condition + session | subject) + (1 + condition + session | item). Treatment contrasts were used as factor coding with "AV" in condition, "pre" in session, and "AC" in group as baseline levels. Interactions are indicated by the symbol ":".

Predictors	Odds Ratios	CI	*p*
(Intercept)	6.47	3.98–10.52	**<.001**
condition [A]	0.08	0.06–0.12	**<.001**
session [post]	0.75	0.55–1.03	.071
group [ST]	0.75	0.45–1.25	.271
age_z	0.92	0.74–1.14	.459
condition [A] : session [post]	1.20	0.81–1.78	.371
condition [A] : group [ST]	1.04	0.66–1.61	.877
session [post] : group [ST]	2.10	1.39–3.15	**<.001**
condition [A] : session[post] : group [ST]	0.59	0.35–0.99	**.047**
**Random effects**			
σ^2	3.29		
$\tau_{00\ \text{subject}}$	0.71		
$\tau_{00\ \text{item}}$	2.85		
$\tau_{11\ \text{subject.condition[A]}}$	0.23		
$\tau_{11\ \text{subject.session[post]}}$	0.07		

τ_{11} item.condition[A]		0.64
τ_{11} item.session[post]		0.06
**Contrasts**		
condition [A]	1	
condition [AV]	0	
session [pre]	0	
session [post]	1	
group [AC]	0	
group [ST]	1	
